# Non allergic gastrointestinal manifestations of hereditary alpha-tryptasemia

**DOI:** 10.3389/falgy.2025.1598309

**Published:** 2025-06-10

**Authors:** Dylan Vainer, Kathryn Peterson

**Affiliations:** ^1^ Department of Internal Medicine, The University of Utah, Salt Lake City, UT, United States; ^2^ Division of Gastroenterology, Hepatology, and Nutrition, The University of Utah, Salt Lake City, UT, United States

**Keywords:** hereditary alpha-tryptasemia, gut brain axis, gastrointestinal disease, mast cell, mastocytosis

## Abstract

Hereditary alpha-tryptasemia (H*α*T) is an autosomal dominant genetic trait characterized by elevated basal serum tryptase due to increased TPSAB1 gene copy numbers. Affecting approximately 4%–6% of the Caucasian population, H*α*T is associated with mast cell-mediated symptoms, including cutaneous reactions, anaphylaxis, and functional gastrointestinal (GI) disorders. While the prevalence of H*α*T in various disorders of gut brain interaction (DGBI)is unknown, individuals with H*α*T exhibit GI disturbances. Mast cells, present throughout the GI tract, release mediators like histamine and prostaglandins, affecting gut motility, secretion, and permeability. Mast cell mediated activation of protease-activated receptors may contribute to gut barrier dysfunction, exacerbating the gastrointestinal symptoms. H*α*T-related GI symptoms are commonly misdiagnosed as other GI conditions, highlighting the need for increased awareness and further research into its pathophysiology and clinical impact. There are no randomized controlled trials on therapy for H*α*T mediated GI symptoms. Current treatment options are based on expert opinion, observational studies, and case reports. Effective therapies parallel those given for clonal mast cell disorders, primarily consisting of antihistamines and mast cell stabilizers. Further research is necessary to delineate the pathophysiology of H*α*T in the gastrointestinal tract so that targeted therapies may be developed. Herein, we aim to describe the pathophysiology, clinical manifestations, diagnostic features, and current/future therapeutic options for patients suffering from H*α*T-mediated GI symptoms.

## Background

Hereditary alpha-tryptasemia (H*α*T), an autosomal dominant genetic trait characterized by elevated basal serum tryptase, results from increased copy number of the TPSAB1 gene encoding alpha-tryptase. It has been reported that up to 4%–6% of the general Caucasian population in the United States have this trait (1). Increased TPSAB1 copy numbers appear to correlate with more mast cell mediated symptoms and multi-system complaints. These manifestations include cutaneous flushing, pruritus, anaphylaxis, and gastrointestinal (GI) symptoms mimicking irritable bowel or disorders of gut brain interaction (DGBI) (2). Currently, the prevalence of H*α*T in disorders of gut brain interaction is unknown. Yet, the release of potent mediators from mast cells may affect secretion and smooth muscle function in the gastrointestinal tract. Thus, GI manifestations of H*α*T likely reflect the effects of mast cell activation in involved tissues.

### Postulated pathophysiology of GI symptoms

Mast cells are present in all segments of the gastrointestinal tract, where via activation of receptors they can release mediators including as prostaglandins and histamine. These mediators can regulate various functions of the GI tract (3). For instance, the release of histamine and prostaglandin D2 leads to the secretion of water and chloride into the intestinal lumen and facilitates intestinal motility. Additionally, as mast cell mediators are released, they sensitize nociceptors in the intestine, contributing to visceral hypersensitivity (4, 5). Mast cells within the muscularis propria interact with the nervous plexi to induce muscle contraction via release of histamine and proteases. Incitement of motor neurons from mast cell degranulation can lead to hypersecretion and increased abnormal peristalsis which can cause cramping and frequent loose bowel movements (6).

Alterations in permeability of the intestinal barrier likely contributes to GI manifestations of H*α*T. One hypothesis regarding gut barrier abnormalities associated with H*α*T implicates protease-activated receptor 2 (PAR-2). This receptor is activated by *α*-tryptase containing mature tryptases; these select tryptases are more prevalent in mast cells from individuals with H*α*T. PAR-2 is highly expressed on intestinal epithelium and involved in intestinal paracellular permeability when activated by tryptase. Gut permeability has been shown to be increased via PAR-2 activation and may contribute to the gut barrier impairment that contributes to the various GI symptoms seen in these patients (4, 7–9).

### Clinical presentations

Clues about possible GI manifestations of H*α*T are derived from both the understanding of effects of mast cell activation on the GI tissues/nerves and from reports of conditions in which mast cells have been identified. GI symptoms of mast cell activation have included abdominal cramping and loose stool, mimicking those of IBS (i.e., DGBI) (10). A recently described condition, mastocytic enterocolitis, may also give clues to the potential manifestations of H*α*T. Similar to diarrhea-predominant IBS, patients with mastocytic enterocolitis present with chronic intractable diarrhea without other alarm features. Patients typically also have unremarkable serological findings and histology other than aggregates of 20 mast cells per high-power field or more in the intestinal mucosal layer (11). In a prior study, 33 out of 47 patients with chronic intractable diarrhea of unclear etiology were determined to exhibit greater than 20 mast cells per high-power field in colonic biopsies. In such cases, mastocytic enterocolitis was diagnosed via biopsies that contained immunostaining for mast-cell tryptase. Unfortunately, no serum tryptase levels were reported at this time. Thus, it is unclear whether these patients suffered from H*α*T.

A similar condition, mastocytosis, with elevated tryptase, has been associated with a myriad of gastrointestinal symptoms which likely parallel those expected from H*α*T (12). Mastocytosis patients may present with non-specific symptoms which may be attributable to mast cell mediator release or from ramifications of high acid output due to associated, excessive histamine release. Thus, similar to mastocytosis, Clinicians should be aware of common conditions such as DGBI, pancreatic insufficiency with or without celiac disease, eosinophilic disorders, and/or infections which present with similar clinical manifestations (Table 1) (2). Table 1 outlines the common clinical manifestations and pertinent workup of H*α*T and other GI diseases that can present with similar symptoms. Common symptoms seen in H*α*T consist of abdominal cramping, dyspepsia, bloating, nausea, and frequent loose stools. Some patients manifest significant malabsorptive diarrhea and peptic ulcer disease.

**Table 1:** Clinical features of hereditary alpha tryptasemia and gastrointestinal diseases with similar manifestations.

Disease	Clinical features	Testing
Hereditary alpha tryptasemia	Abdominal cramping/bloating, constipation, diarrhea, skin flushing, pruritus, and history of recurrent anaphylaxis	Basal serum tryptase >8 ng/ml, tryptase genotyping demonstrating increased copy number of TPSAB1 gene
Irritable bowel syndrome (DGBI)	Chronic abdominal pain with altered bowel habits, no alarm features, improves at night, normal physical exam	Normal CBC, inflammatory markers, and anti-tissue transglutaminase IgA
Inflammatory bowel disease	Bloody diarrhea, fecal urgency, tenesmus, weight loss, fevers	CBC, inflammatory markers, fecal calprotectin, colonoscopy with biopsies
Pancreatic insufficiency	History of cystic fibrosis, chronic pancreatitis, steatorrhea, weight loss	Abdominal CT with findings of chronic pancreatitis, fecal elastase, elevated fecal fat, fat-soluble vitamins
H Pylori	Post-prandial epigastric pain, unexplained iron deficiency	Upper endoscopy biopsy with urease testing, urea breath testing, stool antigen assay
Celiac disease	Gluten food trigger, history of autoimmune disease, abdominal pain in addition to diarrhea with bulky, foul-smelling, floating stools, flatulence, steatorrhea, iron deficiency anemia	Positive tissue transglutaminase IgA, anti-gliadin antibodies, upper endoscopy with duodenal biopsy
Small intestinal bacterial overgrowth	Bloating, chronic watery diarrhea, flatulence	Positive carbohydrate breath test, proximal jejunal aspirate with 10^5^ colony-forming units per ml coliform bacteria
NSAID-induced dyspepsia	Epigastric pain, postprandial fullness, early satiation, history of NSAID use	Upper endoscopy with mucosal erosions, ulcerations, subepithelial hemorrhage
Eosinophilic gastrointestinal disease	History of atopic disease, abdominal pain, nausea, vomiting, early satiety, intestinal obstruction, ascites	Empiric elimination diet, peripheral eosinophilia, upper endoscopy with biopsies of stomach and duodenum
Disaccharidase deficiency	Food triggers, abdominal pain, bloating, fullness, gas, indigestion, cramping	Sucrose breath testing, duodenal endoscopic biopsies with analysis of sucrase, maltase, lactase, and isomaltose levels, trials of food avoidance

GI manifestations of H*α*T are very similar to that of IBS (DGBI), based upon the ROME criteria. The ROME IV criteria were most recently updated in 2016 and are defined by recurrent abdominal pain on average at least one day per week in the past three months with two or more of the following criteria: (1) related to defection (2) associated with change in the frequency of stool and (3) associated with a change in the form of stool (13). There is a high prevalence of presumed DGBI among individuals with elevated basal tryptase levels. A study of 96 individuals with elevated serum tryptase levels found that the prevalence of “IBS” (now with the nomenclature of Disorders of Gut Brain Interaction) as defined by the ROME III criteria was 49%, which is a prevalence of three to five-fold higher than that of the general population (14). Despite this, there is no scientific evidence that supports a relationship between the increased prevalence of mast cells in the GI tract and the symptom severity related to IBS. Currently, the evidence does not suggest that mast cell counts are clinical useful when evaluating patients with non-specific gastrointestinal complaints.

There are several observations that indicate that H*α*T-associated GI symptoms may be distinct from IBS/DGBI. A study of 158 individuals with previously diagnosed IBS/DGBI determined that 5% of Caucasians from this population had H*α*T, which is consistent with the overall prevalence previously described in the general Caucasian population (15). Thus, the functional GI symptoms related to H*α*T may be distinct from DGBI. In another retrospective study of 101 patients with confirmed H*α*T, GI-related complaints were the most common symptoms reported in the review of systems. In this group of patients, abdominal bloating, constipation, diarrhea, and abdominal pain were reported in 90 out of 101 subjects (89%) (16). Despite reported association with DGBI, it remains difficult to differentiate the GI manifestations of H*α*T with that other functional GI disorders. Additionally, without available therapies for these patients which definitively inhibit mast cell activation, it is difficult to define which symptoms can be directed attributed to tryptase in H*α*T.

### Association with eosinophilic gastrointestinal disease (EGID)

Although more data is needed to determine whether a true association with H*α*T exists with EGID, elevated tryptase has been identified in patients with diagnosed eosinophilic gastrointestinal diseases (i.e., Eosinophilic Esophagitis and Eosinophilic Gastroenteritis). Tryptase may encourage the recruitment of eosinophils into tissues and/or incite eosinophil degranulation via cleavage of PAR-2 on eosinophils (17). In fact, injections of tryptase into the peritoneal tissues of animals induce eosinophil infiltration while tryptase inhibitors reduce airway inflammation and eosinophilia in airways (18–20). Thus, if the gastrointestinal tract manifests increased tryptase deposition, eosinophil infiltration may follow, which may result in EGID.

A cohort of 72 patients with eosinophilic esophagitis and gastroenteritis patients demonstrated increased tryptase elevation in a subset (21). Interestingly, the serum tryptase correlated with asthma, urticaria, arthralgia, sinusitis, and increased abdominal symptoms but was not directly related to whether the eosinophilic disease was active or inactive. The serum tryptase remained elevated regardless of disease resolution (21).

Others have reported increased prevalence of celiac disease in H*α*T. In a retrospective cohort study from the University of Toronto of 101 patients with positive genotyping for H*α*T, the prevalence of individuals with celiac disease was 5%, which is significantly higher than that reported in the general population (0.9%). In addition, patients with H*α*T had a significantly higher proportion of positive anti-tTG antibodies compared to the general population (4.9% vs. 0.8%, respectively) (22).

Thus, GI manifestations can be assumed to be related to H*α*T only after all other potential sources of symptoms are excluded (23). Serum tryptase should be measured whenever the cause of DGBI symptoms is elusive or when the symptoms of DGBI do not resolve with therapy. There should a high degree of suspicion for H*α*T when a BST level is found to be greater than 8 μg/L.

### Histopathology

Biopsies of various locations within the GI tract can be used to determine if there are increased numbers of mast cells within the mucosa, however this approach is fraught with error as no specific criteria exist for the diagnosis presently. Prior studies have used a cutoff of 20 mast cells per high-power field in the lamina propria to suggest mastocytic involvement, but this is not based upon current guidelines and may not reflect true mast cell activation in tryptasemia.

Inflammation is likely increased in patients with H*α*T. Evidence of duodenal mast cells along with class-switched memory B cells, indicating low grade inflammation, have been identified in the small intestinal biopsies from a large irritable bowel cohort in which HAT was identified in 5% (15).

Efforts have been made to describe gastrointestinal pathology in H*α*T. In one study, duodenal biopsies were compared between 17 patients with HAT, 15 patients with mast cell activation with low tryptase levels, and 12 controls. H*α*T patients demonstrated increased median count of 30 mast cells/HPF compared to the other cohorts (*P* < 0.004). The mast cells were described as being identified in clusters in several situations (24). In this study, it was suggested that one should to perform stains to identify mast cells in endoscopic biopsies obtained from patients with mast cell activation symptoms who have a baseline serum tryptase >8 ng/ml (24). However, until further data is available, detection of mast cells or tryptase density on GI biopsies is not routine nor informative for patients with suspected H*α*T as discrepancies exist in suggested diagnostic thresholds and the value of enumerating intact mast cells compared to measurement of tryptase density in tissues has not been determined (25).

### Possible therapy for GI manifestations

Current recommended therapies direct effects to reduce the effect of mast cell mediators. Many gastrointestinal symptoms of H*α*T improve with pharmacotherapy that inhibit mast cell meditation. Most of the current treatments are based on the management guidelines for clonal mast cell diseases. Antihistamines, specifically H1 and H2 blockers, are the most common therapies used to treat mast-cell mediated symptoms of the GI tract. H2 antagonists decrease hypersecretion of gastric acid and improve diarrhea and abdominal cramping. Common regimens include famotidine (up to 40 mg BID) (26). Proton pump inhibitors (PPI) may be added to antihistamines in patients with abdominal complaints unresponsive to antihistamines alone. PPI effectiveness may be related to suppression of acid production (27). Cromolyn sodium, an inhibitor of mast-cell degranulation, has been shown to improve GI symptoms in small studies of patients with systemic mastocytosis (28, 29). Leukotriene antagonists (such as montelukast) can be useful as adjunctive therapy to histamine antagonists (30) Table 2.

**Table 2:** Recommended possible pharmacologic therapies for symptom control in adult patients with GI manifestations of H*α*T (29).

Treatment ladder	Drug class	Examples of drugs/recommended dose
1st-line	H2-antagonist	Famotidine 10–20 mg BID Cimetidine 300 mg QID^a^
2nd-line	Proton pump inhibitors	Omeprazole 20 mg/d Pantoprazole 40 mg/d Rabeprazole 20 mg/d
3rd-line	Mast cell stabilizer or leukotriene antagonist	Sodium cromolyn 100–200 mg QID Monteleukast 10 mg po QD
4th-line	Corticosteroid	Prednisone 0.5–1 mg/kg/d starting dose; taper as feasible based on response/tolerance

^a^ The table is recommended initial dose but increases in doses to alleviate symptoms may occur under allergist or gastroenterologist supervision. These are current examples of medications that can be tried. Other medications are currently being studied.

If current therapies fail one may consider using prednisone at a daily dose of 0.5 mg/kg over a two-to-three-week taper, although there are no randomized trials to support this practice (31). Recommended initial doses of the above therapies are delineated in Table 2; increases in doses to alleviate symptoms may occur under allergist or gastroenterologist supervision.

Given that there have been minimal clinical trials in pharmacologic therapies, patients with evidence of H*α*T can consider elimination diets if it is suspected that mast cell activation contributes to symptoms of DGBI (32). A 2-week elimination diet likely can assess symptomatic response. Development of symptoms after reintroducing certain foods suggests the presence of trigger foods. A meta-analysis that reviewed elimination diets in patients with IBS/DGBI suggested that eggs, dairy, wheat, and foods with salicylates or amines worsened symptoms (33). Unfortunately, this study is limited by the lack of testing for tryptase and thus the exact role of elimination diets in H*α*T has yet to be defined. Interestingly, butyrate, a dietary metabolite of fiber, may inhibit mast cell activation via effects on signaling and transcription pathways as well as on calcium influx (34). Thus diets high in fiber, although not studied, may be beneficial. Monitoring of treatment should focus on improving patient symptoms as there are no clinically validated biomarkers to guide therapy.

Therapies targeted towards inhibition of Immunoglobulin E (IgE) or towards direct inhibition of mast cell activation are currently being studied. In retrospective studies the use of omalizumab, which is an IgE monoclonal antibody, was found to reduce incidence of anaphylaxis or urticaria, however there was no evidence that it improved GI manifestations (2). A recent clinical trial of omalizumab in asthmatic patients found that patients with more ß-tryptase-containing alleles did not respond well to omalizumab. Thus, omalizumab may have limited efficacy in mast cell disorders such as H*α*T. This realization has led to the development and subsequent phase II trial of an anti-tryptase monoclonal antibody (NCT04092582), whose results have not yet been published. In addition to tryptase neutralization, which is theorized to improve symptomatic burden in H*α*T, there are other promising targeted therapies that are currently being studied in clinical trials. Monoclonal antibodies that modulate the IL-33 and sialic acid-binding immunoglobulin-type lectins-8 (SIGLEC-8) signaling pathways are also being studied (35). Inhibitors of the bruton's tyrosine kinase pathway are another class of drugs that have been shown to reduce mast cell reactivity in humans (36).

### Evaluation for H*α*T

Due to the protean nature of symptoms associated with H*α*T, it is imperative that a thorough work up be performed to either diagnose or exclude clinically similar conditions (Table 1). As described above, elevated tryptase has been reported in eosinophilic gastrointestinal disorders (EGIDs) and thus it is important to either diagnose or exclude these conditions prior to attributing all symptomatology to H*α*T.

However, patients who present with IBS-type/DGBI symptoms without other potential source of symptoms in addition to other mast cell-related dermatologic (urticaria, flushing, pruritus) or pulmonary (wheezing, dyspnea) complaints should have a serum tryptase level checked to evaluate for mast cell activation disorders. After exclusion of gastrointestinal conditions with similar presentations (or after adequate treatment of these conditions), H*α*T should be suspected. 80 percent of individuals with H*α*T have an elevated basal serum tryptase (BST) level above 11.4 ng/ml, however any level above 8 ng/ml should indicate that H*α*T could be present (37). Patients who are highly symptomatic should also be evaluated for coexisting clonal mast cell disorders such as mastocytosis. One should look for signs that other mast cell mediated conditions are present; lymphadenopathy, hepatosplenomegaly, hematologic cell abnormalities, and eosinophilic tissue infiltration are clues that a coexisting disorder is present. Advanced systemic mastocytosis should be ruled out as mast-cell infiltration of the GI tract can result in significant organ dysfunction and requires treatment with advanced therapies including tyrosine kinase inhibitors and chemotherapeutic agents.

## Conclusion

In conclusion, there is growing scientific consensus that the GI manifestations of H*α*T are a distinct entity from that of functional GI disorders. The summary of evidence demonstrates abnormal immune system activation and gut permeability that are separate from that of the disorders of gut brain interaction. Mast cell clonal disorders and mast cell activation disorders should be taken into consideration when evaluating patients who complain of chronic abdominal symptoms without a clear etiology despite extensive workup—collaboration with Hematology is extremely helpful in discerning the etiology of elevated tryptase. Patients who present with clinical signs and/or symptoms of mast cell activation and have a baseline serum tryptase greater than 8 ng/ml should undergo genetic testing for H*α*T. Currently, treatment focuses on mast cell mediator-directed therapies; however, targeted therapies against tryptase over-expression and other unique mechanistic pathways that reduce mast cell reactivity are currently being evaluated. Future investigation into targeted therapy that neutralizes mature tryptases is promising to yield effective treatments for individuals suffering with GI symptoms related to H*α*T.
